# Processing of Retinal Signals in Normal and HCN Deficient Mice

**DOI:** 10.1371/journal.pone.0029812

**Published:** 2012-01-18

**Authors:** Luca Della Santina, Ilaria Piano, Lorenzo Cangiano, Antonella Caputo, Andreas Ludwig, Luigi Cervetto, Claudia Gargini

**Affiliations:** 1 Department of Physiological Science, University of Pisa, Pisa, Italy; 2 G. B. Bietti Foundation for Ophthalmology, Rome, Italy; 3 Institute of Experimental and Clinical Pharmacology and Toxicology Friedrich-Alexander University, Erlangen, Germany; 4 Department of Psychiatry and Neurobiology, University of Pisa, Pisa, Italy; Dalhousie University, Canada

## Abstract

This study investigates the role of two different HCN channel isoforms in the light response of the outer retina. Taking advantage of HCN-deficient mice models and of in vitro (patch-clamp) and in vivo (ERG) recordings of retinal activity we show that HCN1 and HCN2 channels are expressed at distinct retinal sites and serve different functions. Specifically, HCN1 operate mainly at the level of the photoreceptor inner segment from where, together with other voltage sensitive channels, they control the time course of the response to bright light. Conversely, HCN2 channels are mainly expressed on the dendrites of bipolar cells and affect the response to dim lights. Single cell recordings in HCN1^−/−^ mice or during a pharmacological blockade of I_h_ show that, contrary to previous reports, I_kx_ alone is able to generate the fast initial transient in the rod bright flash response. Here we demonstrate that the relative contribution of I_h_ and I_kx_ to the rods' temporal tuning depends on the membrane potential. This is the first instance in which the light response of normal and HCN1- or HCN2-deficient mice is analyzed in single cells in retinal slice preparations and in integrated full field ERG responses from intact animals. This comparison reveals a high degree of correlation between single cell current clamp data and ERG measurements. A novel picture emerges showing that the temporal profile of the visual response to dim and bright luminance changes is separately determined by the coordinated gating of distinct voltage dependent conductances in photoreceptors and bipolar cells.

## Introduction

Hyperpolarization-activated cyclic nucleotide-gated channels (HCN) are widely expressed in both central and peripheral nervous system where, upon activation by hyperpolarization of an inwardly rectifying current (I_h_), are thought to serve a variety of functions [1]–[2]. An interesting case is the retina where all four HCN channel isoforms (HCN1-4) are expressed differentially [3]–[4] and I_h_ has been measured in both spiking and non-spiking neurons. In rod and cone photoreceptors I_h_ has been characterized with electrophysiological recording techniques [5]–[10]. Expression of the HCN1 and 2 has been recently demonstrated on the dendrites of rod bipolar cells and, correspondingly, an inwardly rectifying current with the properties of I_h_ has been recorded in these neurons [11]. At variance with the heart and with several CNS locations, where HCN are associated to the generation of rhythmic potentials, in the retina they do not seem to cause oscillations, but instead appear to shape the membrane potential fluctuations that encode light stimuli. One of the most striking actions of I_h_ is to generate, along with an ionic conductance named I_kx_, a band-pass filter effect in rod responses to light [8], [12]–[17]. Current-voltage relations and activation properties of whole-cell I_h_ in rods and bipolar cells have been described in some detail but the actual role of the individual HCN isoforms in retinal processing remains unclear.

The functional role of HCN channels has been also approached by non-invasive recordings of the electrical activity of the retina in intact animals [18]. Although the contribution of HCN is poorly reflected in the conventional flash electroretinogram (ERG), it becomes evident in the band-pass profile of the frequency response curves (FRCs) obtained with sinusoidal light stimuli. An HCN blockade with specific organic inhibitors changes the FRCs profile by suppressing the band-pass filter effect [19]. The effect of functional HCN1 channels on the kinetics of the light response of both rods and cones has been recently confirmed by ERG recordings obtained from normal and HCN1 knock-out mice [20]. These results, however, leave open a number of questions on how HCN channels interact with other conductances of the photoreceptor and bipolar cell membrane, nor provide sufficient clues on to whether the different isoforms have distinct functional roles in retinal processing. Insights into these problems may be obtained by measuring the retinal activity in HCN deficient mice models. In this study we investigate the light response of the distal retina in normal and genetically deficient mice for either one of the two most widely expressed isoforms, namely HCN1 and 2. To this purpose we compare ERG and single-cell current clamp measurements in the different mouse models and show that both the HCN1 and HCN2 isoforms, along with the I_kx_ channels and perhaps also other conductance, have a role in setting the temporal properties of the visual response.

## Methods

### Ethics Statement

All the experimental procedures involving animals were carried according to the ARVO Statement for the Use of Animals in Ophthalmic and Vision Research (d.l. 116/92; 86/609/CE). The protocol was approved by the Animal Care Committee of the University Of Pisa, Italy (Protocol. N. 10568, July 25th 2008).Animals were kept in a local facility with water and food ad libitum, under a 12∶12 h light∶ dark cycle with illumination levels below 60 lux. Special care was exercised to limit any suffering and discomfort associated with the experimental procedures that were all conducted under deep anesthesia.

### Animals

Adult HCN1^−/−^, HCN2^−/−^ and littermate controls (HCN^+/+^) were used for immunolabeling, RT-PCR, western blotting analysis, whole cell recordings and ERG experiments. HCN1^−/+^ animals were obtained from The Jackson Laboratory [20], [21], where they are maintained on a 129SvEv background. For experiments, 129SvEv HCN1^−/+^ animals were crossed with C57Bl/6J wild-type mice in order to obtained hybrid HCN1^−/+^. These animals were intercrossed to produce HCN1^−/−^ and HCN1^+/+^ littermates. Genotyping was done by PCR using primer 1F1 (5′-TAATGTTCTCGCAGCCTATG-3′), 2F1 (5′-CCTCAATGAAAACTGCAAGGAGC-3′) and 1R4 (5′-AAGATTGGGCACTACACGCT-3′). HCN2^−/−^ mice have been described previously [22]. HCN2^−/+^ animals on a hybrid 129Sv/C57Bl/6J background were intercrossed to generate HCN2-deficient and control animals. Genotyping was done by PCR using primers 14 F (5′-GGTCCCAGGCACTTCCATCCTTT-3′), 156 R (5′-GGAAAAATGGCTGCTGAGCTGTCTC-3′) and 16 F (5′-CAGCTCCCATTTGCCCTTGTGC-3′).

All the experimental procedures involving animals were carried according to the ARVO Statement for the Use of Animals in Ophthalmic and Vision Research (d.l. 116/92; 86/609/CE). Animals were kept in a local facility with water and food ad libitum, under a 12∶12 h light∶ dark cycle with illumination levels below 60 lux.

### Immunohistochemistry

Adult mice were deeply anaesthetized with urethane 20% W/V in 0.9% saline before eye-enucleating. The retinas in the eyecup were immersion-fixed for 20 min in 4% paraformaldehyde in 0.1 M phosphate buffer saline (PBS, pH 7.4) at room temperature and then washed 3 times for 10 min in PBS. Tissue was cryoprotected in scalar dilution (10, 20, and 30%) of sucrose in PBS. Eyecups were then included in Tissue Tek Optimal Cutting Temperature (OCT) compound (Miles incorporated, Elkhart NL) and sectioned at −20°C into a cryostat. Serial sections of 18 µm in thickness were collected on super-frost plus slides (Fluka Biochemika).

Sections were washed 3 times for 10 min in PBS and then incubated in 1% bovine serum albumin (BSA) and 0.3% Triton-X 100 in PBS 0.1 M for 45 min in order to block unspecific binding and induce membrane permeability. Sections were incubated for 48 h at 4°C with primary antibodies (polyclonal anti-HCN1, anti-HCN2, 1∶100 dilution, Sigma-Aldrich; monoclonal anti-PKC 1∶100, Sigma-Aldrich) diluted in 1% BSA and 0.03% Triton-X 100 in PBS. Sections were washed in PBS and incubated in secondary antibodies (anti-mouse or anti-rabbit conjugated with Alexa Fluor 488 or with Alexa Fluor 568, 1∶200 Molecular Probes) diluted in 1% BSA in PBS for 2–3 h at room temperature, washed in PBS and cover slipped with Vectashield (Vector Laboratories). Retinal sections were visualized with a confocal microscope equipped with a krypton-argon laser (TCS-NT, Leica Microsystem, and Wetzlar Germany); files were processed with image manipulation software (Photoshop CS2, Adobe Systems Incorporated, San Jose CA).

### mRNA expression analysis

Total RNA was extracted from mouse retina using RNeasy Fibrous Tissue kit (Qiagen). For RT- PCR, 1 µg of total RNA was retro transcribed with both random hexamer and oligo (dT) primers using the Quant Tect Reverse Transcription Kit (Qiagen). Conventional RT-PCR was used to examine the expression of HCN1-2. We used the following primer sets: HCN1: forward: AGGTTAATCAGATACATACACC, reverse: GAGTGCGTAGGAATATTGTTTT, 231-bp amplicon; HCN2: CGGCTCATCCGATATATCCA, reverse: AGCGCGAACGAGTAGAGCTC, 230-bp amplicon; PCR conditions: 15 min 95°C; 40 cycles: 10 s 95°C, 40 s 60°C, 40 s 72°C. The identity of PCR products was verified by agarose gel electrophoresis [23]. All lanes were loaded with the same amount of reaction product (5 µl) to obtain a semi-quantitative evaluation of expression. Cyclophilin served as an internal standard, forward: GGCTCTTGAAATGGACCCTTC, reverse: CAGCCAATGCTTGATCATATTCTT, 91-bp amplicon [24].

### Perforated-patch clamp recordings

Isolation of the dark adapted retinas (>3 hrs) and slicing were performed with a naked eye under dim illumination in the far red (LEDs with peak emission at 720 nm; Chen Guang Optoelectronic, Jiangmen City China). Following anesthesia by i.p. injection of 2,2,2-tribromethanol (Sigma-Aldrich, St. Louis MO; 15 mg/kg), each retina was rapidly extracted through a corneal incision into cold O_2_/CO_2_ bubbled AMES medium integrated with sodium bicarbonate (Sigma-Aldrich), and the vitreous delicately removed with forceps. A retina was laid vitreal side down on filter paper, made to adhere to it by weak transmural suction, and slices of 250 µm thickness were cut with a manual tissue chopper (mod. 600; The Vibratome Company, St. Louis MO). Once secured within the recording chamber slices were visualized in the near infrared (LED peak emission at 780 nm) with a CCD camera attached to an upright microscope (Leica Microsystems, Wetzlar Germany) while being continuously perfused with the same AMES medium at a temperature of 24°C. HCN inhibition was obtained by adding ***3*** µM ***ivabradine*** (Institut de Recherches Internationales Servier, Courbevoie, France) to the perfusing medium [25]. Pipettes for perforated patch recording (6–9 MΩ) were pulled with a P-97 (Sutter Instrument, Novato CA) and filled with a solution containing in mM 90 K aspartate, 20 K_2_SO_4_, 15 KCl, 10 NaCl, 5 Pipes, corrected to a pH of 7.20 with KOH/HCl. The back-filling solution also contained 0.4 mg/ml Amphotericin-B (Sigma-Aldrich) pre-dissolved in DMSO at 60 mg/ml. Recordings were made with an Axopatch 1D amplifier, low-pass filtered at 500 Hz and digitized at 5 kHz (200 Hz/1 kHz during input impedance measurement), and acquired by pClamp 9 software (both from Axon Instruments, Foster City CA). Membrane potentials were not corrected for the liquid junction and Donnan potentials [26], due to the large uncertainties involved in their estimate in perforated patch recordings with Amphotericin B. Full field light stimuli were delivered to the preparation by an LED (OD520; Optodiode Corp., Newbury Park CA) mounted beside the objective turret and conditioned through an optical band-pass filter (509–519 nm) and a neutral density filter (0.9 log units). The photon flux density reaching the recording chamber as a function of LED drive was measured separately with an optical power meter (Model 1815-C; Newport, Irvine CA). The neuronal frequency-response characteristics was explored by delivering, in current clamp, a sinusoidal current stimulus of 50 s duration, modulated in frequency continuously and monotonically between 0.1 and 30 Hz referred to in the literature as a ZAP stimulus [27]. We modified it in order to give equal representation in the time domain to each frequency decade. A full description of the current stimulus, and of the analysis procedure used to obtain neuronal impedance profiles is given in Cangiano et al. [11].

### Electroretinogram (ERG)

The general procedure for animal preparation, anesthesia, ERG recording, light stimulation and data analysis has been previously described in detail in Della Santina et al. [19]. Briefly: ERGs were recorded in complete darkness via coiled gold electrodes making contact with the moist cornea. A small gold plate placed in the mouth served as both reference and ground. HCN inhibition was induced by subcutaneous injections of 12 mg/kg ivabradine. Responses were amplified differentially, band-pass filtered at 0.1 to 500 Hz, digitized at 12.8 kHz by a computer interface (LabVIEW 6.1; National Instruments, Austin, TX) and stored on disc for processing. Responses to flashes were averaged with an interstimulus interval ranging from 60 s for dim lights to 120 s for the brightest flashes.

The full field illumination of the eyes was achieved via a Ganzfeld sphere 30 cm in diameter, whose interior surface was coated with a highly reflective white paint. Two stimulus patterns were adopted: brief flashes that generated the typical ERG response (a- and b-waves) and sinusoidal time varying luminance stimuli eliciting periodic responses.

### Flash stimuli

An electronic flash unit (SUNPAK B3600 DX) generated a stimulus whose energy decayed in time with a τ = 1.7 ms. A short-wavelength band-pass filter, 7.5 nm half bandwidth (Spindler and Hoyer, Gőttingen, Germany), was used, which gave a scotopic effective λ of 492 nm. Because the maximal energy of the band-pass filtered flashes was not sufficient to elicit saturating a-wave responses, these were obtained by delivering flashes of white light whose scotopic efficacy was evaluated according to Lyubarsky and Pugh [28]. The estimated maximum retinal luminance was 7.6×10^5^ Φ (Photoisomerisation Rod^−1^) per flash. Calibrated neutral density filters were used to attenuate the intensity of the flashes.

### Time varying sinusoidal stimulation

Sinusoidal changes in luminance at various temporal frequencies and modulation depth were generated by a light-emitting diode (LED) source (peak wavelength: λ = 520). The luminance of sinusoidal stimuli is expressed as:
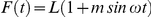
where “L” is the mean luminance and “m” is the contrast.

A light stimulus unit developed in our laboratory generated sinusoidal temporal patterns [29]. For all these experiments, we used a stimulus intensity corresponding to a mean retinal luminance of 38.79 Φ per second and a contrast value of 85%.

### Analysis of ERG Responses to Sinusoidal Light Stimulation

The recorded signals were averaged in synchrony with the stimulus luminance periodicity and a discrete Fourier analysis was performed to estimate amplitude and phase of the first harmonic. Corrections were made to allow for the amplifier's filter properties. The frequency response curves (FRCs) reported in the results were obtained by plotting the amplitude of the first harmonic as a function of the temporal frequency.

## Results

### Transcript and protein expression and immunohistochemistry match expectations for the HCN1^−/−^ and HCN2^−/−^ mice

**Figure 1:**
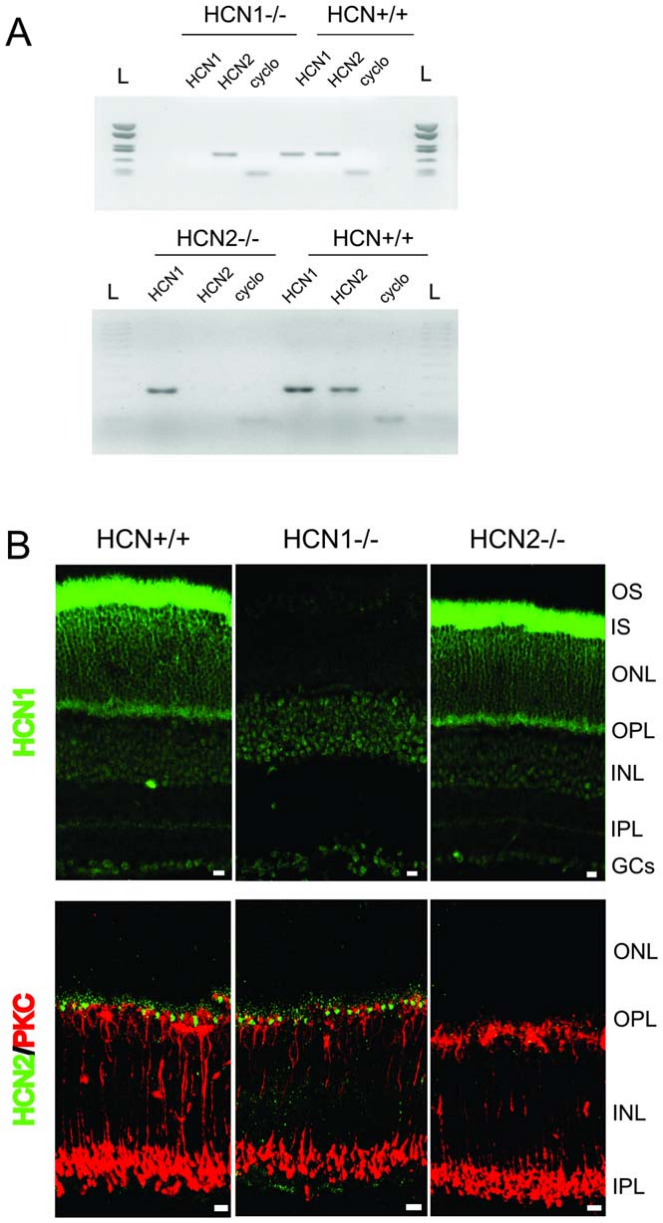
Transcript expression and immunohistochemistry of HCN channels. A: HCN channel 1–2 mRNA expression in murine retina. The amount of HCN amplicons is compared to cyclophilin expression. B: Confocal images of retinal sections immunolabeled with rabbit polyclonal antibodies (green fluorescence) specific for HCN1 (upper panel) and HCN2 (bottom panel) in HCN^+/+^, HCN1^−/−^ and HCN2^−/−^ mice. In addition to immunolabeling with the antibody for HCN2 (bottom panel), the retinas were also stained with an antibody against PKC, a specific marker for rod bipolar cells (red fluorescence). Scale bars, 10 µm.

Retinal transcripts of HCN1-2 isoforms detected by RT-PCR from HCN^+/+^ and HCN1^−/−^ or HCN 2^−/−^ mice are shown in Fig. 1A. The mRNA of HCN1-2 subunits is expressed in retinas of HCN^+/+^ littermates. As expected, the signal for the HCN1 or 2 transcripts is missing in the respective HCN knockout mice. Confocal images of immunofluorescence-stained trans retinal sections are shown in Fig. 1B. Both HCN1 and 2 proteins are expressed across the retinal layers of normal mice, showing that HCN1 isoforms are mainly located at the inner segments of photoreceptors whereas HCN2 are distributed postsynaptically and in particular on the dendrites of rod bipolar cells [11]. See, however, that a much weaker staining for both isoforms is also observed in other retinal regions and especially at the inner plexiform layer. Retinal sections from HCN1^−/−^ and HCN2^−/−^ do not show any specific staining for HCN1 or HCN2 proteins, respectively. Collectively, these data are evidence that the two knockout mouse lines used in our study are valid animal models. We thus shifted to electrophysiology to investigate the functional role of the HCN1 and HCN2 isoforms in the outer retina.

### HCN1 channels sharpen the initial “nose” in the rod voltage response to bright flashes but are not required for its expression

We investigated the role of the HCN channels in rod bright flash responses with patch clamp recordings obtained in dark-adapted mouse retinal slices. It has long been assumed that the initial sharp transient of voltage responses of rods to bright flashes, commonly referred to as nose, reflects the activation by membrane hyperpolarization of a current flowing through the HCN channels. A role of I_h_ in generating the initial nose was first proposed for lower vertebrate rods [30] and later predicted, but never actually tested, also in mammals [8], [31]. We performed this test in the rods of HCN^+/+^, HCN1^−/−^ and HCN2^−/−^ mice.

**Figure 2:**
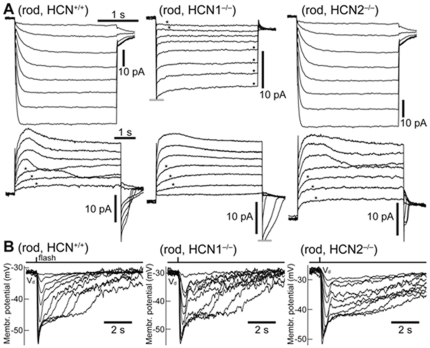
Voltage-gated currents and flash responses of rods in HCN^+/+^, HCN1^−/−^ and HCN2^−/−^ mice. **A (records above):** currents recorded in rods in response to hyperpolarizing voltage clamp steps from a holding potential of −53 mV, to −60/−67/−74/−81/−88/−95/−102/−109 mV, and depolarization to −65 mV. A slow-activating I_h_ current was present in HCN^+/+^ and HCN2^−/−^, but not in HCN1^−/−^ rods. In the latter, the absence of I_h_ left an instantaneous inward rectifying current (dots). **A (records below):** currents recorded in the same rods in response to depolarizing voltage steps from −64 mV, to −57/−50/−43/−36/−29/−22/−15 mV, and repolarization to −60 mV. A slow-activating I_kx_ current was present in all rods (stars). **B:** photovoltage responses of dark adapted rods to flashes of green light (514 nm) of increasing strength, covering over 3-log units (range 0.2–780 photons/µm^2^). The fast initial nose following bright flashes was present in both normal and HCN deficient rods. Flashes were delivered at the rods' apparent dark membrane potential (V_d_). Baselines are aligned to each other (max shift 2 mV). Records are averages of several sweeps and are ‘box car’ filtered with a window of 20 ms. Data obtained at 24°C.

We measured in voltage-clamp the membrane current changes evoked by step hyperpolarization or depolarization. The results are illustrated in Fig. 2A where it is seen that hyperpolarizing steps activated I_h_ in the rods of HCN^+/+^ (n = 19) and HCN2^−/−^ (n = 3) mice, but not in those of HCN1^−/−^ mice (n = 5). In HCN1^−/−^ rods, instead, an inward-rectifying current with instantaneous kinetics was present, activating negative of −74/−81 mV (n = 5). HCN1^−/−^ rods, in contrast to HCN^+/+^ and HCN2^−/−^, also did not display tail currents following the hyperpolarizing steps suggesting that tail currents are entirely due to the deactivation of I_h_. Based on tail currents, I_h_ in HCN^+/+^ rods activates negative of −60/−67 mV. These results are consistent with the notion that HCN1 is the sole isoform expressed by rods.

**Figure 3:**
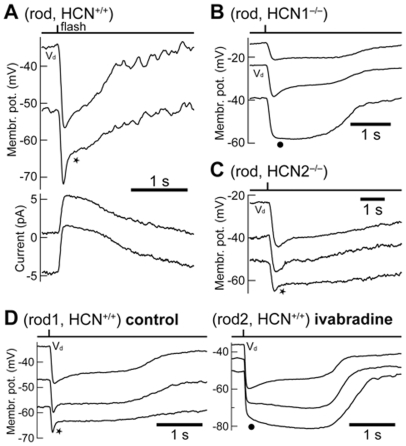
Rod responses to bright flashes in HCN^+/+^, HCN1^−/−^ and HCN2^−/−^ mice at different membrane potentials. **A**: bright flashes (109 photons/µm^2^) were delivered in HCN^+/+^ rods at the dark membrane potential (V_d_) and at a more hyperpolarized potential maintained by constant current injection (upper traces). The same flashes were also delivered in voltage clamp while holding the rod at −40 and −50 mV, respectively. In current clamp the nose was more prominent at the hyperpolarized potential (star), but it was always absent in voltage clamp. **B**: in contrast to HCN^+/+^, in HCN1^−/−^ rods the nose (flash strength 195 photons/µm^2^) was present at V_d_ but disappeared at more negative potentials. **C**: in a rod from an HCN2^−/−^ animal, hyperpolarization speeded up the nose (54 photons/µm^2^) similarly to what observed in normal HCN^+/+^ mice. **D**: pharmacological blockade of I_h_ with 3 µM ivabradine (right traces) abolished the nose (236 photons/µm^2^) at hyperpolarized (dot) but not at depolarized potentials. Compare this with the behavior of a rod recorded in the same preparation prior to perfusion with ivabradine and stimulated with the same flash (left traces). Records are averages of several sweeps and are ‘box car’ filtered with a window of 20 ms. Data obtained at 24°C.

**Figure 4:**
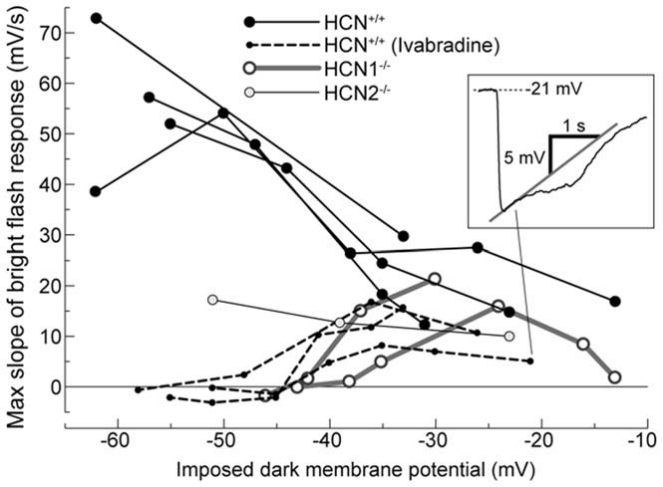
Summary graph of the degree of nose in the bright flash response in HCN^+/+^, HCN1^−/−^ and HCN2^−/−^ mice, as a function of dark membrane potential (V_d_). The nose was quantified by taking the maximum slope of photovoltage trajectory in the first second following the flash (inset). The dark membrane potential was imposed by constant current injection. Inspection of the data shows that I_h_ is entirely responsible for generating the nose at hyperpolarized potentials, while at depolarized potentials this role is played by another current, presumably I_kx_. There may exist a range of V_d_ within which both mechanisms cooperate to quicken the bright flash response of rods.

Bright flashes were delivered in current-clamp at the apparent dark membrane potential (V_dark._) of rods. Note that this value is likely to be more depolarized than the unperturbed V_dark_, due to shunting introduced by the finite seal resistance of the patch pipette on the cell's membrane [11]. As expected, the rods of HCN^+/+^ (n = 22) and HCN2^−/−^ (n = 4) animals expressed a typical nose in response to bright flashes (Fig. 2 B). Surprisingly, this was also true of rods that lacked I_h_, which were recorded in HCN1^−/−^ mice (n = 3; Fig. 2 B). We thus investigated the origin of the rod nose in the experiments summarized in Fig. 3 A–D, in which bright flashes were delivered while holding the cell membrane at different potentials. Fig. 3 A shows that the nose, while present in the photovoltage of HCN^+/+^ mice (upper traces), was absent in the photocurrent at all potentials (lower traces). It must then arise from the action of voltage-gated currents downstream of phototransduction. In HCN^+/+^ (n = 4) and HCN2^−/−^ (n = 2) mice the nose became sharper and more pronounced with hyperpolarization into the range of activation of I_h_ (Fig. 3 A/C/D, star). On the contrary, in HCN1^−/−^ rods (n = 2), hyperpolarization had the effect of suppressing the nose (Fig. 3 B, dot). We further strengthened the causal link between the lack of I_h_ in HCN1^−/−^ rods and the disappearance of the nose upon membrane hyperpolarization, by pharmacologically blocking this current in HCN^+/+^ retinas. Perfusion with 3 µM ivabradine blocked I_h_ in rods, as confirmed by hyperpolarizing steps delivered in voltage clamp (n = 2, not shown) and in agreement with a recent study [25]. In ivabradine rods displayed a nose in response to bright flashes delivered at V_dark_ but not upon hyperpolarization (n = 3; cf. Fig. 3 D control/ivabradine: two rods from the same retina recorded prior/during washing with ivabradine, respectively; the control and treated rods had to be separate cells, due to the relatively short duration of rod seals). Fig. 4 summarizes these data, by plotting the maximum slope of the bright flash response in the first second after the flash, as a function of the membrane potential at which the flash was delivered (i.e. a V_dark_ imposed by constant current injection). Positive values indicate the presence of a nose as a rapid depolarization immediately after the peak response, whereas values near zero correspond to a plateau without the nose. All rods expressed a nose at V_dark_ values more depolarized than −35/−40 mV, independently of the presence of the HCN1, HCN2 or I_h_. On the other hand, when I_h_ was absent in rods (HCN1^−/−^, or HCN^+/+^ with ivabradine) the nose was not present for V_dark_ more hyperpolarized than −40/45 mV. These observations indicate that more than one current can contribute to the nose of the rod response to bright flashes: I_h_ plays a greater role at hyperpolarized potentials, with other currents acting in a more depolarized range. By examining the outward currents expressed by rods in both normal and HCN deficient mice, a candidate was identified having slow kinetics, activating upon depolarization positive of −57/−50 mV, and not showing inactivation (Fig. 2 A). This current, which is partially active at V_dark_, has properties matching those of the I_kx_ current [17] and may explain the presence of the nose in HCN1^−/−^ rods. I_kx_ is generally thought to play a marginal role with saturating flashes [32], but these data show that the relative contribution of I_h_ and I_kx_ will depend on the unperturbed state of the rod, including its true value of V_dark_.

### Multiple HCN isoforms control the temporal properties of outer retina

**Figure 5:**
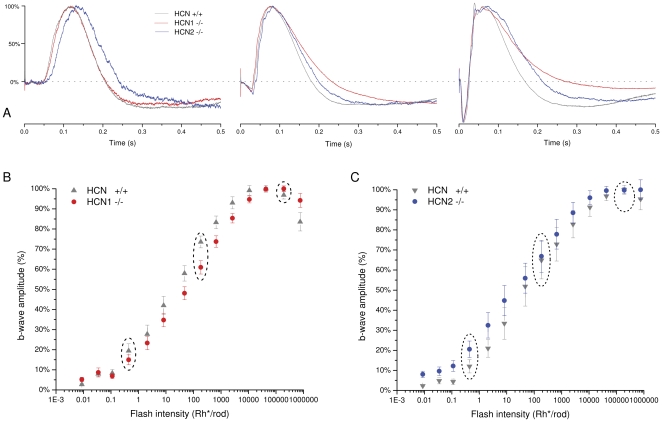
ERG response to flashes of increasing intensity. A: averaged ERG responses of increasing light intensity in the HCN^+/+^ (gray, n = 18), HCN1^−/−^ (red n = 18) and HCN2^−/−^ (blue, n = 10). Dim, intermediate and bright flash intensities are shown in the left, middle and right panel, respectively. B–C: collected data of the b-wave peak amplitude as a function of the flash intensity in HCN^+/+^, HCN1^−/−^ and HCN2^−/−^, relative amplitudes were normalized at their maximum value. The intensity of the flash is expressed as a number of photoisomerizations per rod (Φ) per flash. The dotted ovals in B indicate the dim, intermediate and bright flash responses illustrated in A.

It has been recently shown that the functional impact of the HCN channels on the early stages of retinal processing may be effectively investigated by ERG recordings [19]. The ERG response to flashes of increasing intensity obtained from normal and HCN deficient mice and collected from all the experiments is reported in Fig. 5. The records in A are averaged responses (the number of experiments is indicated in the figure) to dim, intermediate and bright luminance flashes recorded from HCN^+/+^, HCN1^−/−^ and HCN2^−/−^ mice. In B the normalized amplitude of the b-wave is plotted as a function of light intensity. The most relevant, feature that characterizes the flash response of HCN deficient animals are the kinetics profiles of the b-wave which varied with flash intensity. Compared to the time course of the HCN^+/+^ b-waves, in HCN2^−/−^ these responses are slowed and delayed to a greater extent in the range of dim flashes, while in HCN1^−/−^ the largest difference is recorded in response to bright flashes. In all cases the response of genetically deficient mice is slowed down mainly in the decay phase. These results are consistent with the notion that HCN1 channels are mainly expressed at the inner segments of photoreceptors and HCN2 on the dendrites of on bipolar cells (see Discussion).

**Figure 6:**
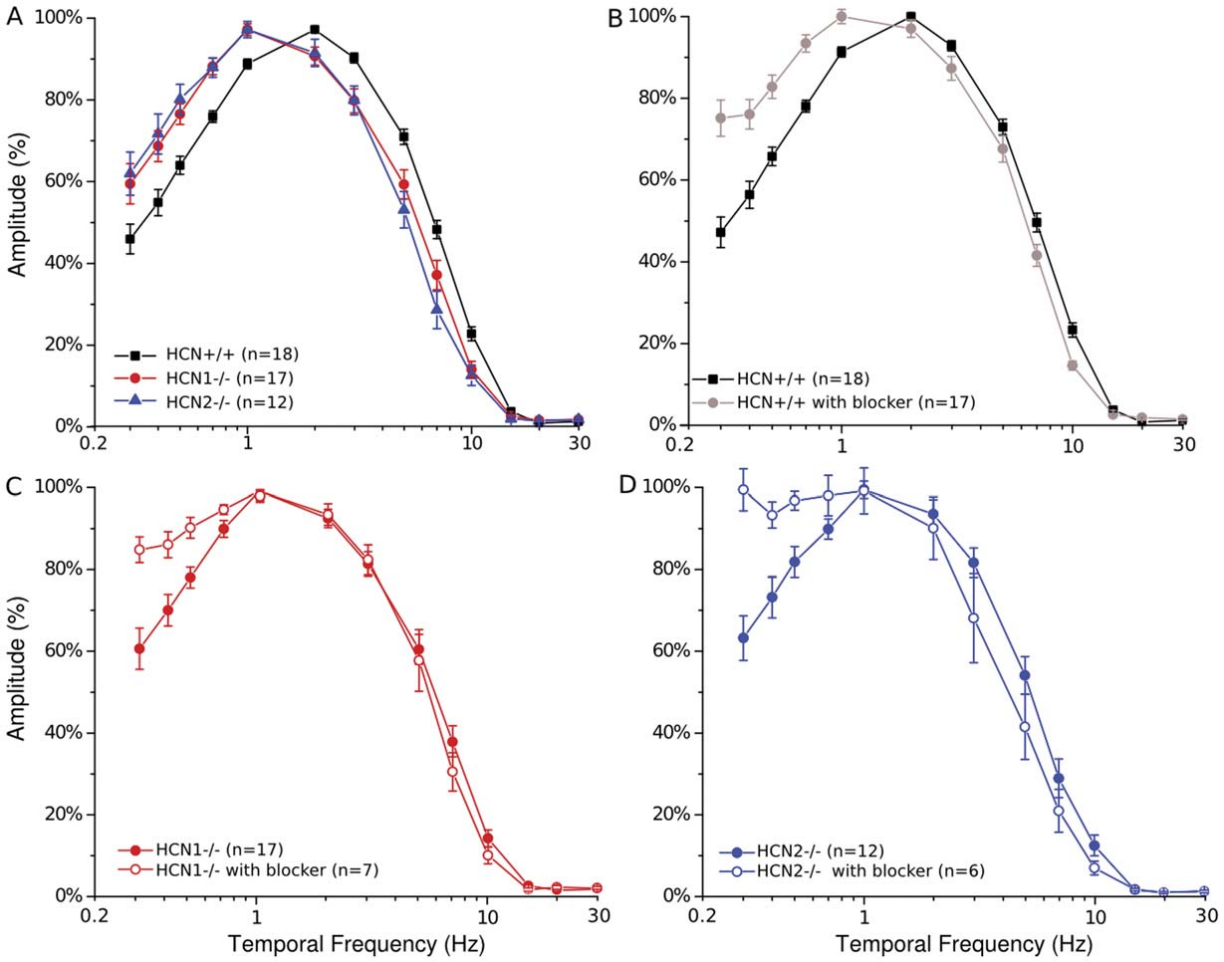
ERG response to sinusoidal time varying luminance stimuli. FRCs obtained by sinusoidal modulation of a mean luminance equivalent to 38.79Φ in HCN^+/+^ (n = 15), HCN1^−/−^ (A, n = 17) and HCN2^−/−^ (B, n = 12) before, and after blocker injection (12 mg/kg; n = 7/n = 6 respectively for HCN1^−/−^ and HCN2^−/−^). Relative amplitude was normalized at their resonance peak. Stimulus contrast, 85%; vertical bars = SEM.

The temporal response properties of the outer retina can be better appreciated by examining its FRC profile obtained with the ERG, complemented by a single cell analysis in photoreceptors. The results of the ERG experiments are illustrated in Fig. 6. Responses from normal mice are compared with those from HCN1^−/−^ and HCN2^−/−^ in Fig. 6A. In both lines of HCN deficient mice it is seen that the resonance peak and cut-off are shifted to lower temporal frequencies than those in the wild type. Nonetheless, in HCN deficient mice the FRCs retain a band-pass character. A pharmacological inhibition of the HCN-mediated I_h_ current by ivabradine (panels B–D) causes a generalized reduction of the FRC band-pass profile in all mice models.

**Figure 7:**
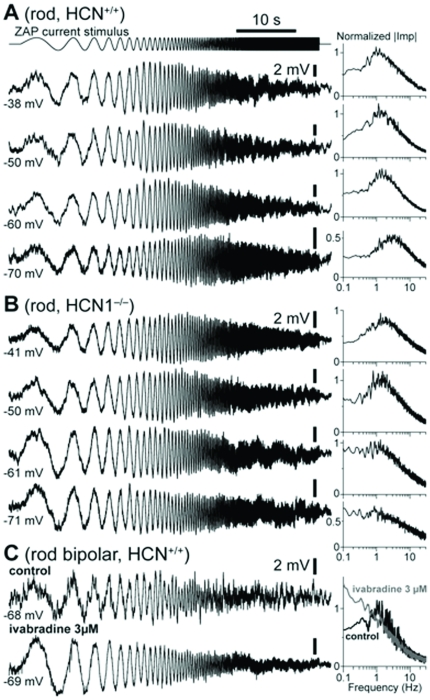
Sinusoidal current injections explore the FRC of HCN^+/+^ and HCN1^−/−^ rods. **A/B**: ZAP stimuli consisting of small amplitude sinusoidal modulated current stimuli (0.1 and 30 Hz, duration 50 s) were delivered in current clamp in dark adapted rods, at various potentials by constant current injection. Voltage responses are shown below, together with the corresponding normalized input impedance profiles. Resonance is expressed in both mouse lines, although in HCN1^−/−^ it is entirely abolished when the membrane is hyperpolarized below −55/−60 mV. **C**: The same protocol delivered in rod bipolars highlights their resonant membrane properties, which disappeared upon perfusion with the specific HCN inhibitor ivabradine 3 µM. Records are averages of several sweeps. Data obtained at 24°C.

The frequency-response characteristics of single rod photoreceptors were determined by delivering, in current-clamp, a sinusoidal current stimulus of 50 s duration, modulated in frequency continuously and monotonically between 0.1 Hz and 30 Hz (ZAP stimulus, see Methods). By this approach one measures the input impedance of the neuron's membrane. The results of these measurements show a prominent band pass profile in both normal (Fig. 7 A) and HCN1-deficient rods (Fig. 7 B) when the membrane potential was held at −50 mV, or more depolarized. Similarly to its effect on the shape of the bright flash response (see above), hyperpolarization abolished the band pass profile in HCN1^−/−^ rods (n = 2), but not in normal rods (n = 2). It is thus clear that I_h_ is not the only current able to shape the frequency response of rods and that, depending on their actual membrane potential, the relative contribution of I_h_ and of other currents such as I_kx_ will vary. Based on these and other experiments shown in Figs. 3 and 4, I_h_ will contribute more at more hyperpolarized membrane potentials. This may explain why the FRCs measured with the ERG maintained a degree of band-pass behavior even during phamacological blockade of I_h_ (Fig. 6 B–D). Fig. 7 C shows the responses to a ZAP stimulus of a rod bipolar cell sitting at V_dark_ in control and after I_h_ inhibition by ivabradine. It is seen that in control conditions the cell impedance displays a typical band pass profile, but after HCN inhibition this is converted into low-pass with a much lower cut-off, near or below the lowest tested frequency. These effects are reminiscent of those observed on the FRCs of the ERG response. These results taken together strongly suggest that the sinusoidal modulation of light backgrounds, delivered during the ERG recordings, hyperpolarized rods to a level at which both I_h_ and the other resonance-endowing currents were partially activated.

## Discussion

The present study explores the relative contribution of two HCN channel isoforms expressed in the outer retina to the temporal integration of visual signals. Both HCN1 and HCN2 were found to enhance the band-pass response of the retina measured with the ERG, but while HCN2 acts on dim luminance changes, HCN1 comes into play at brighter light levels. This functional organization matches the expectations from the morphological distribution of the two isoforms, as well as from single cell data presented here and in previous studies. The picture that emerges from these results is novel and shows how the gating of different voltage dependent conductances interacts in photoreceptors and bipolar cells to set the temporal profile of the visual response to dim and bright luminance changes. This is the first instance in which single cell light responses from control and HCN^−/−^ mice are compared with the integrated full field ERG response from intact animals. The comparison reveals a high degree of correlation for data from current clamp and ERG measurements.

One of the most striking features of the voltage response of retinal rods to the onset of bright lights is the initial transient (or “nose”) in the hyperpolarizing response, which has been described in retinas of all animal species. For decades there has been a general consensus on the idea that this fast nose was due to the activation of Ih, the voltage dependent current that flows through HCN channels. I_kx_ has long been recognized as having a role limited to the shaping of dim light responses, while only I_h_ would come into play under bright light [32]. The observation that a fast nose is also present in HCN1^−/−^ mice rods from where no I_h_ has been recorded imposes a revision of this notion. The new picture that now emerges, supported by the rod recordings shown in this study, is that at least two distinct conductances including G_h_ and G_kx_ activated at different membrane potentials play a role in shaping the time course of the rod bright light photovoltage. In a recent study in salamander [10] the role of I_h_ in setting the time course of photoreceptor flash responses was studied with a pharmacological blockade of I_h_. The authors reported that the nose in the rod bright flash response was completely abolished without I_h_, but did not test the impact of membrane potential, which we show here to be of critical importance in determining the relative contribution of I_h_ and I_kx_. In addition, since an adequate control appears not to have been performed, it is possible that the relatively high concentration of the antagonist ZD7288 used in their study (50 µM; cf. 1 µM in Cangiano et al. [11]) inhibited not only I_h_, but also other currents including I_kx_.

The present study also contributes to clarify the functional significance of the different HCN isoforms expressed in the outer retina. Convergent evidence from immunolabelling and electrophysiological studies strongly indicate that HCN1 isoforms are mainly expressed at the inner segments of the photoreceptor (Fig. 1C and 2A) whereas HCN2 are distributed on the dendrites of rod bipolar cells ([11] and Fig. 1C). In HCN1^−/−^ mice the b-wave of the ERG in response to bright flashes is slower than in normal controls, while in HCN2^−/−^, appreciable kinetics changes also occur in the temporal course of the response to dim flashes (see Fig. 5). This is consistent with the notion that the temporal profile of the b-wave at dim and bright luminance is controlled by two distinct processes operating, respectively, at the bipolar cell level, through HCN2, and at the receptor level through HCN1. These results show that HCN affect mainly the kinetics of the b-wave with little effect on that of the a-wave. This is not surprising because the leading edge of this response is known to reflect the current suppression by light at the outer segments of the visual cell *with little influence from the inner segment currents* [33]. The HCN seem also to have no effect on the light sensitivity, but they do reduce the absolute amplitude of both a- and b-waves of the ERG. There is not an obvious explanation for this effect which may reflect a reduction of the dark current associated with the absence of HCN whose mechanisms is not understood.

In a previous study on rats we have shown that the most evident effect of the HCN pharmacological inhibition can be observed on the profile of the FRC of the ERG. Here we confirm this observation also in mice and show that in either HCN1^−/−^ or HCN2^−/−^ the FRCs behave as though partial pharmacological blockade of HCN was induced, thus causing attenuation of the normal band-pass profile. An almost full suppression of the band-pass profile may then be obtained by pharmacological inhibition of the residual HCN still expressed in HCN2^−/−^ or HCN1^−/−^ respectively (Fig. 6).

The membrane impedance of normal, HCN-deficient rods and rod bipolar cells are in substantial agreement with the FRCs of the ERG response. An important implication of this finding is that the gating properties of the HCN channels in photoreceptors and bipolar cells may also be inferred from non invasive ERG recordings. It is important to note, however, that the data from single cells reflect the filtering properties of their membrane suggesting that the impact of the HCN on the visual signals at the retinal output is bound to be determined also by the cascade of stages where the channels operate. Accordingly, the ERG's b-wave, whose main determinants are the rod bipolar cells, must reflect the operation of the second stage. It seems therefore reasonable to assume that the impact of HCN on processing of visual information would be further enhanced in the subsequent stages of the visual system including those in the retina and in the central pathways.
